# Human Cytomegalovirus IE2 86 kDa Protein Induces STING Degradation and Inhibits cGAMP-Mediated IFN-β Induction

**DOI:** 10.3389/fmicb.2017.01854

**Published:** 2017-09-26

**Authors:** Jung-Eun Kim, Young-Eui Kim, Mark F. Stinski, Jin-Hyun Ahn, Yoon-Jae Song

**Affiliations:** ^1^Department of Life Science, Gachon University, Seongnam, South Korea; ^2^Department of Molecular Cell Biology, Samsung Biomedical Research Institute, Sungkyunkwan University School of Medicine, Suwon, South Korea; ^3^Department of Microbiology, Carver College of Medicine, University of Iowa, Iowa City, IA, United States

**Keywords:** HCMV, IE86, STING, IFN, cGAMP

## Abstract

Stimulator of interferon genes (STING) is a critical signaling molecule in the innate immune response against DNA viruses by either directly sensing intracellular DNA or functioning as an adaptor molecule to activate the type I interferon (IFN) signaling pathway. We determined the functional interaction between STING and human cytomegalovirus (HCMV). A cDNA library containing 133 HCMV ORFs was screened to identify viral genes that inhibit STING-induced IFN-β promoter activation. Among the screened ORFs, UL122, which encodes the immediate-early 2 86 kDa (IE86) protein, strongly abolished STING-induced IFN-β promoter activation. Interestingly, IE86 protein facilitated the proteasome-dependent degradation of STING and inhibited 2′3′-cGAMP-mediated induction of *IFNB1* and *CXCL10*. Taken together, this study demonstrates the existence of a post-translational regulation of STING by HCMV IE86 protein.

## Introduction

Stimulator of interferon genes (STING), also known as TMEM173, ERIS, MITA and MPYS, is a critical signaling molecule that plays a key role in the type I IFN signaling pathways in response to DNA virus infection (reviewed in Ishikawa et al., 2009). STING contains four N-terminal transmembrane domains, a dimerization domain and a C-terminal tail. The C-terminal tail of STING serves as a signaling platform for recruiting various signaling molecules to activate the type I IFN response (reviewed in Burdette and Vance, 2013). In response to intracellular DNA derived from viruses, STING interacts with tumor necrosis factor receptor (TNFR)-associated factor (TRAF) family member-associated NF-κB activator (TANK) binding kinase 1 (TBK1). Transcription factors including interferon regulatory factors (IRFs) and NF-κB are activated and induce the expression of type I IFNs and other IFN-stimulated genes (ISGs) (Liu et al., 2015).

STING is regulated by its dimerization, translocation from the endoplasmic reticulum (ER) through the Golgi to the perinuclear location and post-translational modifications such as phosphorylation or ubiquitination (reviewed in Burdette and Vance, 2013). STING has been proposed to function as a direct DNA sensor for cyclic dinucleotides generated by cyclic GMP-AMP synthase (cGAS) (Burdette et al., 2011; Ablasser et al., 2013) or an adaptor protein that is activated by several DNA sensors including interferon-gamma-inducible protein 16 (IFI16) and DExD/H-box helicase 41 (DDX41) upon infection with DNA viruses such as herpes simplex virus 1 (HSV-1), Kaposi’s sarcoma-associated herpesvirus (KSHV), human papillomavirus (HPV), adenovirus and HCMV (reviewed in Ma and Damania, 2016).

The cGAS-STING pathway is critical for activating the type I IFN pathway upon HCMV infection in primary human umbilical vein endothelial cells (HUVEC) and monocytic leukemia cell line THP-1 (Lio et al., 2016; Paijo et al., 2016). Disruption of STING expression in HUVEC cells using the CRISPR/Cas9 system enhances HCMV replication (Lio et al., 2016). In human fibroblasts, the IFI16-STING pathway has been implicated in detecting HCMV DNA and activating the type I IFN pathway (Gariano et al., 2012; Li et al., 2013). Although IFI16 knockdown (KD) enhances HCMV replication (Gariano et al., 2012), a recent study indicates that cGAS, but not IFI16, is required for the STING signaling pathway in human fibroblast upon HCMV infection (Diner et al., 2016).

Although STING plays a pivotal role in DNA virus infection-induced innate immune responses, a regulatory mechanism(s) for STING employed by HCMV protein(s) has not been extensively elucidated. Other viruses have been reported to employ effective mechanisms to counteract the STING pathway (reviewed in Ma and Damania, 2016). For example, HSV-1 ICP27 protein interacts with the STING-TBK1 complex and inhibits type I IFN expression (Christensen et al., 2016), and STING KD in cells derived from normal tissues results in higher HSV-1 titer (Kalamvoki and Roizman, 2014). During the course of HCMV infection, protein levels of several signaling components in the type I IFN pathway including STING are gradually down-regulated (Weekes et al., 2014). A recent study indicates that HCMV tegument protein UL82 inhibits the STING signaling pathway (Fu et al., 2017). It was proposed that UL82 protein interacts with STING and iRhom2, disrupts the STING-iRhom2-TRAPβ complex and inhibits the trafficking of STING from the ER to the perinuclear region (Fu et al., 2017).

In the present study, by screening a HCMV-Towne cDNA library, we found that HCMV UL122 encoding the immediate-early 2 86 kDa (IE86) protein most effectively reduced STING-induced IFN-β promoter activation. Potent inhibition of STING-induced IFN-β promoter activity by IE86 protein was expected because IE86 protein inhibits HCMV-induced IFN-β production by interfering with NF-κB binding activity to the IFN-β promoter (Taylor and Bresnahan, 2005, 2006). Interestingly, we also found that the protein levels of STING were significantly reduced in cells expressing IE86 protein. In addition to transcription factors for IFN-β promoter activation, IE86 protein may target STING to inhibit the type I IFN pathway. Therefore, we investigated the regulation of STING protein stability by HCMV IE86 protein in this study.

## Materials and Methods

### Cells and Viruses

The maintenance and propagation of primary human foreskin fibroblast (HFF) and HEK293T cells were described previously (Stinski, 1976; Kang et al., 2013). Recombinant lentiviral and retroviral vectors were generated by using Lenti-X^TM^ lentiviral expression system and retroviral gene transfer and expression system, respectively, according to the manufacturer’s directions (Clontech). The propagation of HCMV–Towne was described previously (Stinski, 1976; Meier et al., 2002). Standard plaque assay was performed to determine viral titers (Isomura et al., 2005). Propagation and purification of replication-defective E1a^-^, E1b^-^ and E3^-^ adenovirus vector expressing IE72 (Ad-IE72), IE86 (Ad-IE86), green fluorescent protein (GFP) (Ad-GFP) or tetracycline transactivator (Ad-Trans) were described previously (Murphy et al., 2000). The transgene expression with the adenovirus vector system was induced by Ad-Trans (Murphy et al., 2000).

### Reagents, Transfection and Reporter Gene Assays

Proteasome inhibitors, MG132 and epoxomicin, and their vehicle DMSO were purchased from EMD Millipore. Lysosome inhibitor, chloroquine, and cycloheximide were purchased from Sigma–Aldrich and Fisher Scientific, respectively. Cyclic [G(2′5′)pA(3′5′)p] (2′3′-cGAMP) was purchased from InvivoGen. Omicsfect^TM^ for transient transfection was used according to the manufacturer’s directions (Omics Biotechnology). The luciferase assay was performed as described previously (Bari et al., 2011).

### Plasmid Constructs

The shRNA sequences for human STING (hSTING) to generate pLKO.1-STING shRNA-a and -b were obtained from the genetic perturbation platform (GPP) at Broad Institute (TRCN0000163296 and TRCN0000161052, respectively). pLKO.1 scramble shRNA was a gift from David Sabatini (Addgene plasmid #1864) (Sarbassov et al., 2005). To generate a retroviral vector expressing C-terminal Myc-tagged hSTING (pLHCX-hSTING-myc), STING-myc fragment was amplified from pCMVsport6-hSTING (kindly provided by Dr. Kisa Sung, University of Pittsburgh) using PCR with the following primers: 5′-GCAAGCTTGCCATGCCCCACTCCAGCCTGCAT-3′ and 5′-GGCATCGATTCACAGATCCTCTTCTGAGATGAGTTTTTGTTCAGAGAAATCCGTGCG-3′. The PCR products were digested with HindIII and ClaI (New England Biolabs) and ligated into the pLHCX vector (Clontech). pENTR-hSTING vector was generated from pCMVsport6-hSTING, and hSTING was cloned into pEF-based destination vector from the pENTR-hSTING using LR clonase^TM^ enzyme mix (Invitrogen). National center for biotechnology information (NCBI) reference sequence number for hSTING used in this study is NM_198282.1. 6X-myc-IE86 wild type (WT) or deletion mutants were cloned into pCS3-MT (with a 6X-myc tag) -based destination vector from the pENTR-IE86 WT or deletion mutants using LR clonase^TM^ enzyme mix (Invitrogen) as described previously (Park et al., 2007). IE72, IE86 and UL82 were cloned into pEF-GST-based destination vector from the pENTR-IE72, pENTR-IE86 or pENTR-UL82 using LR clonase^TM^ enzyme mix (Invitrogen). pEF-Bos TRIF-FLAG was a gift from Kate Fitzgerald and Tom Maniatis (Addgene plasmid # 41550) (Fitzgerald et al., 2003).

### HCMV cDNA Library

A HCMV-Towne ORF library in the pENTR vector (Invitrogen) was described previously (Kim E.T. et al., 2014). One hundred thirty-three HCMV-Towne ORFs were transferred to the destination vector pDEST-12.2 from pENTR vector using LR clonase^TM^ enzyme mix according to the manufacturer’s directions (Invitrogen).

### Yeast Two-Hybrid Assays

Yeast AH109 (MATa) cells were transformed with plasmid (Trp+) expressing the GAL4-DNA-binding domain (DBD)-STING fusion protein. Y187 (MATa) cells were transformed with plasmid (Leu+) expressing the GAL4-activation domain (AD)-HCMV ORF fusion proteins. Transformants were selected on plates lacking tryptophan or leucine. Trp+ and Leu+ transformants were mated with each other on complete YPD plates and diploid cells (a/α) were selected on plates lacking both tryptophan and leucine. Cells expressing bait and prey that interact with each other grow on plates that lack tryptophan, leucine, and histidine and express β-galactosidase. Cells expressing GAL4-DBD-STING and GAL4-AD only were used as a negative control. For rapid in site assays for β-galactosidase production, a 5-bromo-4-chloro-3-indolyl-β–galactopyranoside (X-Gal) filter assay was used. For quantitative assays, β-galactosidase production was measured using *o*-nitrophenyl-β–galactopyranoside (ONPG) assays. Yeast strains, media for yeast growth, methods for yeast transformation, and β-galactosidase assays were all as described previously (Ahn et al., 2001).

### Western Blot Analysis

Cells were harvested, fractionated and transferred onto nitrocellulose membranes as described previously (Kim et al., 2015). Antibodies to STING (D2P2F), phospho-TBK1 (D52C2) and LC3B (D11) were purchased from Cell Signaling Technology. An anti-FLAG M2 antibody (#200474) was purchased from Agilent technologies. Antibodies to tubulin (B-5-1-2) and TBK1 (AOW9) were purchased from Sigma–Aldrich and EMD Millipore, respectively. Anti-HCMV IE (CH160) antibody was purchased from Virusys. Antibodies to IE86 (12E2), c-Myc (9E10) and GST (B-14) were purchased from Santa Cruz Biotechnology. Secondary peroxidase-labeled anti-mouse or anti-rabbit immunoglobulin G antibodies were purchased from Jackson ImmunoResearch. The signal intensity of protein bands were quantitated using Image Lab^TM^ software (Bio-Rad Laboratories).

### Quantitative PCR

The isolation of total DNA and analysis of HCMV replication using quantitative PCR (qPCR) were performed as described previously (To et al., 2014). The isolation and reverse-transcription of total RNA and analysis of mRNA expression using quantitative reverse transcription PCR (qRT-PCR) were performed as described previously (Kim J.E. et al., 2014) with the following primers : UL122, 5′-ACCATGCAGGTGAACAACAA-3′ and 5′-CATGAGGAAGGGAGTGGAGA-3′; UL44, 5′-GCTGTCGCTCTCCTCTTTCG-3′ and 5′-TCACGGTCTTTCCTCCAAGG-3′; UL83, 5′-GCAGCCACGGGATCGTACT-3′ and 5′-GGCTTTTACCTCACACGAGCATT-3′; hSTING, 5′-AGGAGGAAAAGGAAGAGGTTACTGT-3′ and 5′-TCTTGGGACATCGTGGAGGTA-3′; IFNB1, 5′-ATGACCAACAAGTGTCTCCTCC-3′ and 5′-GCTCATGGAAAGAGCTGTAGTG-3′; CXCL10, 5′-TCCACGTGTTGAGATCATTGC-3′ and 5′-TCTTGATGGCCTTCGATTCTG-3′; GAPDH, 5′-CATGAGAAGTATGACAACAGCCT-3′ and 5′-AGTCCTTCCACGATACCAAAGT-3′.

## Results

### Effect of STING on HCMV Replication in HFF Cells

**FIGURE 1:**
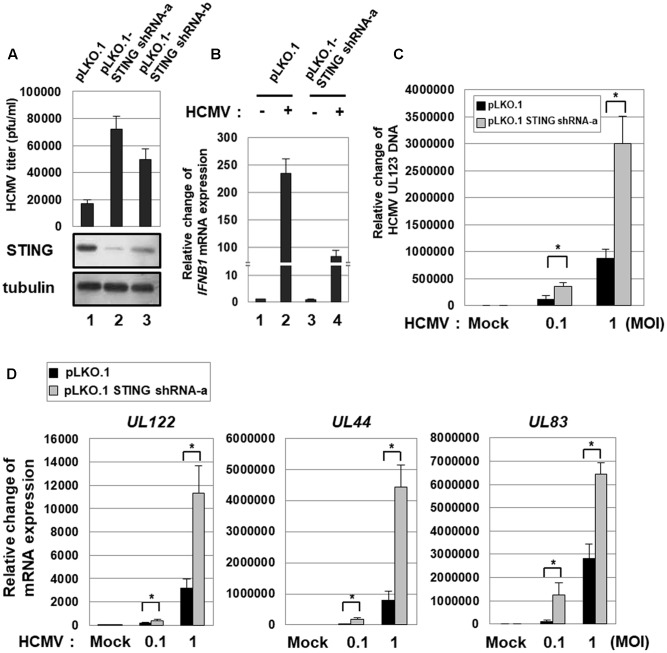
STING KD enhances HCMV replication. **(A)** HFF cells stably transduced with either pLKO.1-scramble or pLKO.1-STING shRNAs (a and b) were infected with HCMV-Towne, and, at 7 days after infection, the viral titer was determined using plaque assays. The KD level of STING protein was determined by western blot analysis. **(B)** HFF cells stably transduced with either pLKO.1-scramble or pLKO.1-STING shRNA-a were either mock-infected or infected with HCMV-Towne at 1 MOI, and relative amounts of *IFNB1* transcripts were measured using qRT-PCR at 6 h after infection. **(C,D)** HFF cells stably transduced with either pLKO.1-scramble or pLKO.1-STING shRNA-a were either mock-infected or infected with HCMV-Towne at 0.1 or 1 MOI. At 5 days after infection, **(C)** the relative amount of HCMV DNA was analyzed by qPCR using primers specific for the viral UL123 gene, and **(D)** the mRNA levels for HCMV UL122 (IE), UL44 (E) and UL83 (L) genes were analyzed by qRT-PCR analysis. Real-time PCR data shown here represent three independent experiments ±*SD*. The asterisk (^∗^) denotes a significant difference between samples, which was determined by the *P*-value of a two-sample *t*-test (*P* < 0.05).

HFF cells stably transduced with either pLKO.1-scramble or two different pLKO.1-STING shRNAs (a and b) were infected with HCMV-Towne, and, at 7 days after infection, the viral titer (pfu/ml) was determined using plaque assays. In cells stably transduced with pLKO.1-STING shRNA-a and -b, the level of STING protein was significantly reduced by 78 and 61%, respectively (**Figure 1A**). Compared with pLKO.1-scramble shRNA-transduced cells, the viral titer was increased 4.3-fold and 2.9-fold in cells transduced with pLKO.1-STING shRNA-a and -b, respectively (**Figure 1A**). Since STING KD was more efficient with pLKO.1-STING shRNA-a than -b, HFF cells stably transduced with pLKO.1-STING shRNA-a were used for the following experiments. In STING KD HFF cells, HCMV infection-induced expression of *IFNB1* transcripts was significantly reduced by 2.8-fold compared with control cells (**Figure 1B**, compare lane 4 with 2). The residual expression of *IFNB1* transcripts induced by HCMV infection in STING KD cells was possibly mediated by the remaining STING protein and/or other innate immune signaling pathways such as Toll-like receptor (TLR) 2 and 9 (**Figure 1B**, compare lane 4 with 1) (Juckem et al., 2008; DeFilippis et al., 2010; Gariano et al., 2012).

To quantitatively analyze the level of HCMV replication in HFF cells stably transduced with pLKO.1-STING shRNA, cells were infected with the HCMV-Towne at low (0.1 pfu/cell) or high (1 pfu/cell) multiplicity of infection (MOI), and the relative amount of viral DNA was measured by qPCR using primers specific for UL123 at 5 days after infection. In STING KD HFF cells, HCMV replication was significantly increased by 2.9-fold at low MOI and 3.4-fold at high MOI compared with control cells (**Figure 1C**).

The levels of UL122 (IE), UL44 (E, early) or UL83 (L, late) transcripts were also determined by qRT-PCR. STING KD induced the mRNA levels of HCMV IE, E and L genes by 2-, 8-, and 10-folds, respectively, at low MOI or by 3.6-, 5.6- and 2.3-folds, respectively, at high MOI (**Figure 1D**). These data indicate that STING KD up-regulates the expression of HCMV lytic genes and induces HCMV replication.

**FIGURE 2:**
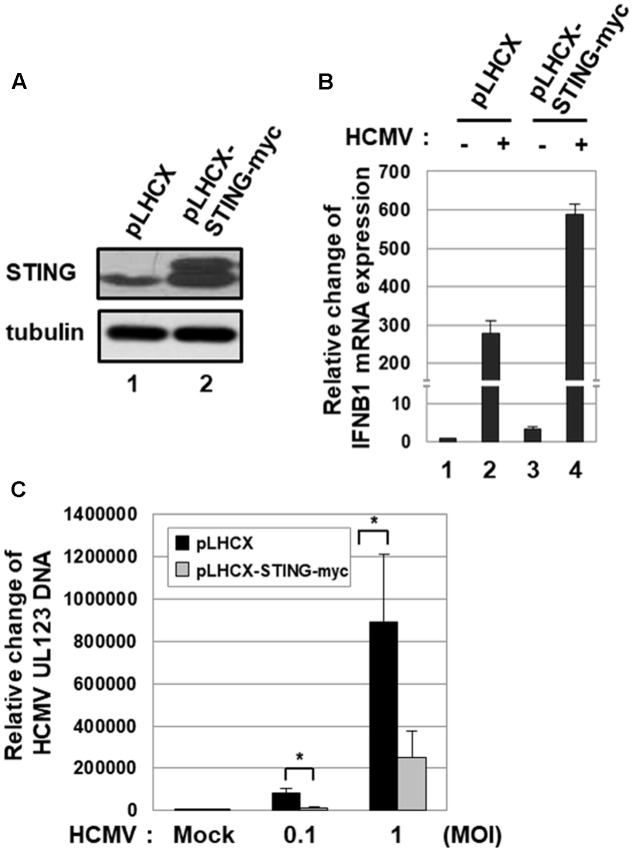
Ectopic expression of STING inhibits HCMV replication. **(A)** HFF cells were stably transduced with pLHCX or pLHCX-STING-myc, and ectopic expression of STING protein was determined by western blot analysis. **(B)** HFF cells stably transduced with pLHCX or pLHCX-STING-myc were either mock-infected or infected with HCMV-Towne at 1 MOI, and relative amounts of *IFNB1* transcripts were measured using qRT-PCR at 6 h after infection. **(C)** HFF cells stably transduced with pLHCX or pLHCX-STING-myc were either mock-infected or infected with HCMV-Towne at 0.1 or 1 MOI for 5 days. The relative amount of HCMV DNA was analyzed by qPCR using primers specific for the viral UL123 gene. Real-time PCR data shown here represent three independent experiments ±*SD*. The asterisk (^∗^) denotes a significant difference between samples, which was determined by the *P*-value of a two-sample *t*-test (*P* < 0.05).

To further determine the effect of STING on HCMV infection, HFF cells stably transduced with either pLHCX- or pLHCX-STING-myc retroviral vector were generated (**Figure 2A**). Ectopic expression of STING protein induced the level of *IFNB1* transcripts 3.3-fold (**Figure 2B**, compare lane 3 with 1). In HFF cells transduced with pLHCX-STING-myc retroviral vector, HCMV infection-induced *IFNB1* expression was further induced 2.1-fold compared with control cells (**Figure 2B**, compare lane 4 with 2). To quantitatively analyze the level of HCMV replication in HFF cells stably transduced with either pLHCX- or pLHCX-STING-myc retroviral vector, the relative amount of viral DNA was measured by qPCR using primers specific for UL123. In HFF cells over-expressing STING, HCMV replication was significantly reduced by 86.7% at low MOI (0.1 pfu/cell) and 71.9% at high MOI (1 pfu/cell) compared with control cells (**Figure 2C**). Taken together, these data suggest that STING plays an important role in limiting HCMV replication.

### Screening of HCMV cDNA Libraries to Identify Viral Genes That Inhibit STING-Induced IFN-β Promoter Activation

**FIGURE 3:**
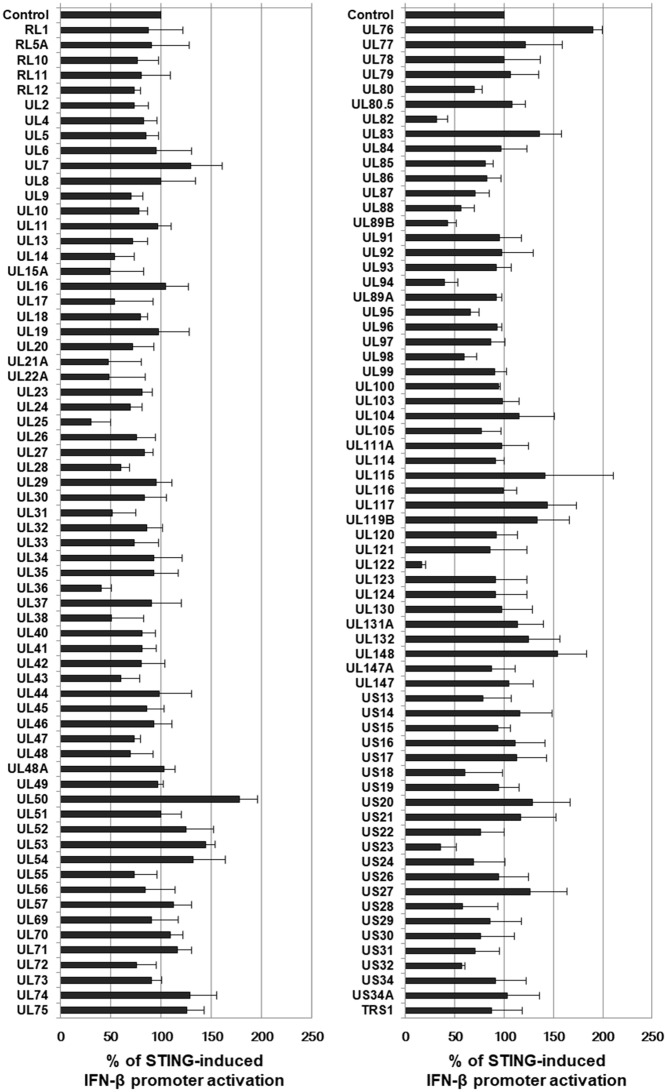
Screening of HCMV cDNA libraries to identify genes that down-regulate STING-induced IFN-β promoter activation. HEK293T cells were co-transfected with the vector expressing STING, IFN-β promoter-driven firefly luciferase reporter and control *renilla* luciferase reporter plasmids plus either pDEST-12.2 or pDEST-12.2 expressing cDNAs encoding 133 HCMV-Towne ORFs. After 24 h, luciferase activity was measured using a dual luciferase assay system. IFN-β promoter-driven luciferase activity was expressed in RLU by normalizing firefly luciferase activity with constitutive *renilla* luciferase activity. To determine the effect of HCMV-Towne ORFs on STING-induced IFN-β promoter activation, RLU in cells co-transfected with the vector expressing STING and pDEST-12.2 vector expressing HCMV-Towne ORF was divided by that in cells co-transfected with control vector and pDEST-12.2 vector expressing HCMV-Towne ORF. To analyze the relative luciferase activity, STING-induced luciferase activities without HCMV-Towne ORF were set to 100%. Luciferase data shown here represent three independent experiments ±*SD*.

To identify HCMV genes that interfere with STING-induced type I IFN response, a cDNA library containing 133 HCMV-Towne ORFs was screened using IFN-β promoter-driven luciferase reporter. HEK293T cells, which do not express detectable STING protein (Diner et al., 2013), were co-transfected with the vector expressing STING, control *renilla* luciferase reporter and IFN-β promoter-driven firefly luciferase reporter plasmids plus expression vectors for 133 HCMV-Towne ORFs. In HEK293T cells, ectopic expression of STING enhanced IFN-β promoter-driven luciferase activity 7–fold. To determine the effect of HCMV-Towne ORF on STING-induced IFN-β promoter activation, relative light unit (RLU) in cells co-transfected with the vector expressing STING and a vector expressing HCMV-Towne ORF was divided by that in cells co-transfected with control vector and a vector expressing HCMV-Towne ORF. To analyze the relative luciferase activity, STING-induced luciferase activities without HCMV-Towne ORF were set to 100%. Among the screened HCMV-Towne ORFs, UL25, UL36, UL82, UL89B, UL94, UL122 and US23 reduced STING-induced IFN-β promoter activation by greater than or equal to 50% (**Figure 3**). HCMV UL122 encoding IE86 protein most effectively reduced STING-induced IFN-β promoter activation by 87% (**Figure 3**).

**FIGURE 4:**
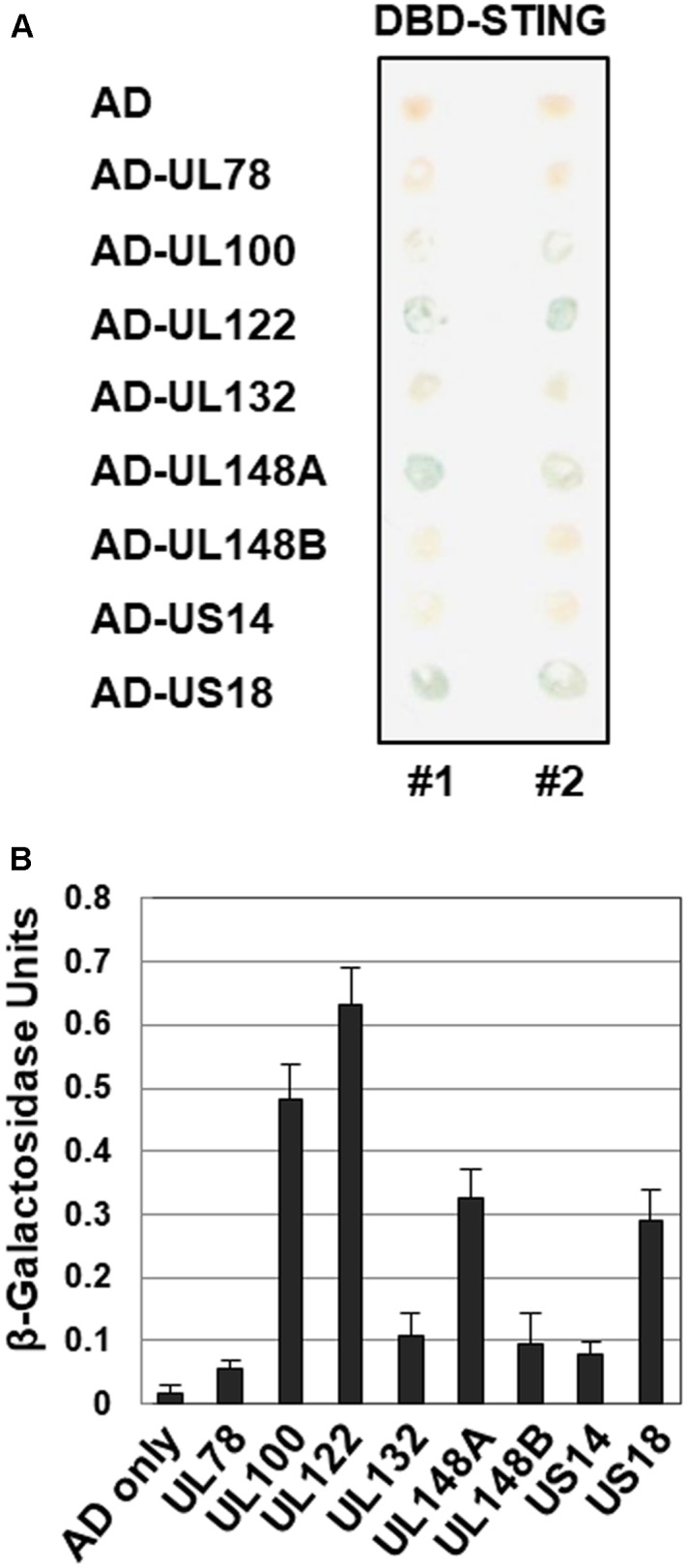
Interaction of STING with HCMV proteins in yeast two-hybrid interaction assay. **(A)** The ability of cells expressing GAL4-DBD-STING and GAL4-AD-HCMV ORF to produce β-galactosidase was assessed using X-gal filter assays. **(B)** The levels of β-galactosidase produced in yeast cell lysates were also quantitatively measured using ONPG as a substrate.

The interaction between STING and HCMV-Towne ORFs was also investigated by a yeast two-hybrid assay as previously described (**Figure 4**) (Ahn et al., 2001). Interestingly, Gal-4 DBD fused to STING interacted strongly with a Gal-4 AD fusion with HCMV UL122 yielding blue colonies in β-galactosidase filter assays after 30 min incubation in yeast two-hybrid assays (**Figure 4A**). Among the screened HCMV-Towne ORFs, UL122 exhibited the strongest interaction with STING (**Figure 4B**). However, Gal-4 AD fusion with HCMV UL25, UL36, UL82, UL89B, UL94 and US23, that reduced STING-induced IFN-β promoter activation, was less sensitive for the detection of β-galactosidase. In addition to HCMV UL122, Gal-4 DBD fused to STING interacted with a Gal-4 AD fusion with HCMV UL78, UL100, UL132, UL148A, UL148B, US14 and US18 (**Figure 4**).

### HCMV IE86 Mediates the Proteasome-Dependent Degradation of STING

**FIGURE 5:**
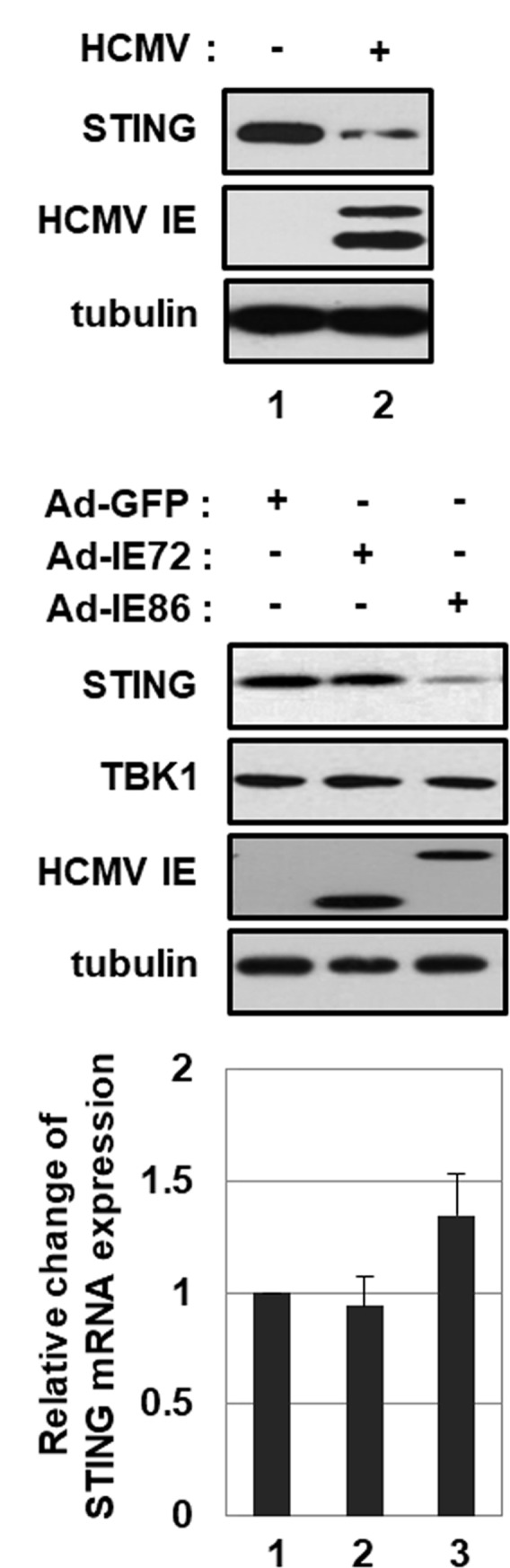
HCMV IE86 protein reduces the level of STING protein. **(A)** HFF cells were either mock-infected or infected with HCMV-Towne. At 24 h after infection, cells were harvested, and equal amounts of cell extracts were subjected to western blot analysis with antibodies to STING, HCMV IE and tubulin. **(B,C)** HFF cells were transduced with 10 pfu per cell of either Ad-GFP plus Ad-Trans, Ad-IE72 plus Ad-Trans or Ad-IE86 plus Ad-Trans and incubated for 48 h. **(B)** Cells were harvested, and equal amounts of cell extracts were subjected to western blot analysis with antibodies to STING, TBK1, HCMV IE and tubulin. **(C)** The level of STING mRNA was analyzed by qRT-PCR analysis. Real-time PCR data shown here represent three independent experiments ±*SD*.

As previously reported (Weekes et al., 2014; Fu et al., 2017), HCMV infection reduced the level of endogenous STING protein in HFF cells (**Figure 5A**, compare lane 2 with 1). Since IE86 protein inhibits STING-induced type I IFN promoter activation, the effect of IE86 protein on STING expression was determined. HFF cells were transduced with 10 pfu per cell of Ad-IE72, Ad-IE86 or Ad-GFP. At 48 h after transduction, the level of STING protein was determined by western blot analysis. Interestingly, the protein level of STING, but not TBK1, was significantly reduced by 4.8-fold in Ad-IE86-transduced cells compared to Ad-GFP-transduced cells (**Figure 5B**, compare lane 3 with 1). The mRNA level of STING was not attenuated in cells transduced with Ad-IE86 (**Figure 5C**, compare lane 3 with 1). Compared to IE86 protein, IE72 protein had no effect on the level of STING protein (**Figure 5B**, compare lane 2 with 1).

**FIGURE 6:**
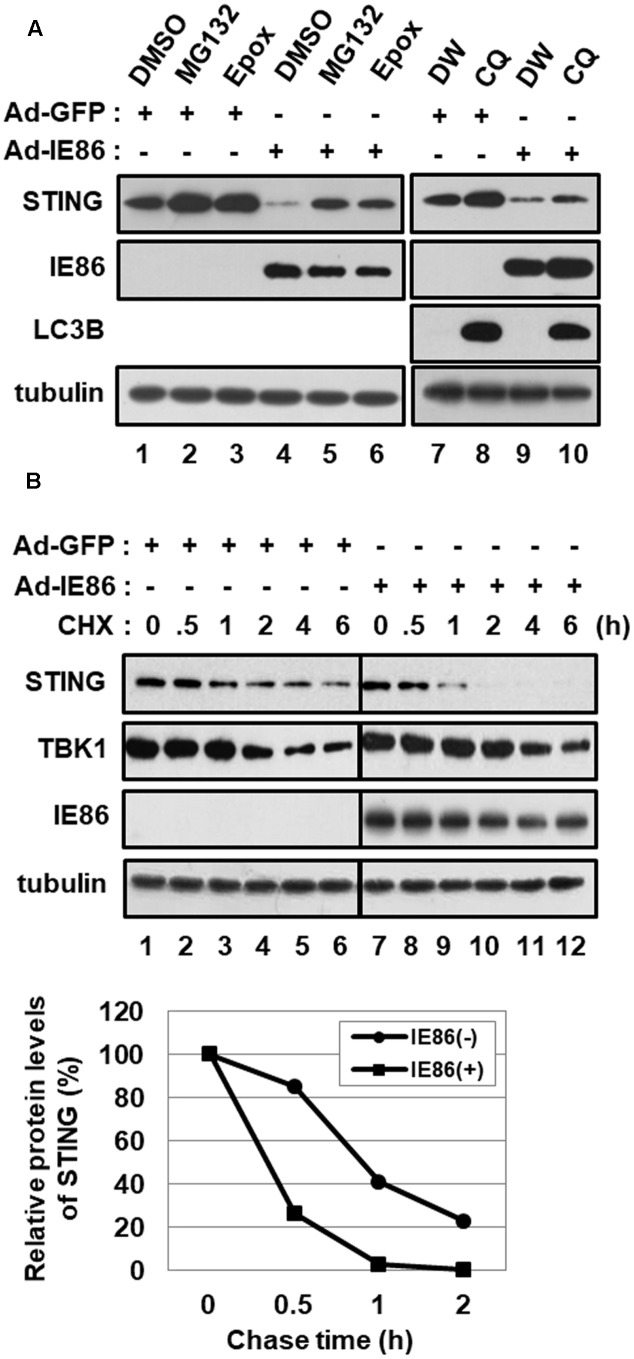
HCMV IE86 protein induces the proteasome-dependent degradation of STING. HFF cells were transduced with 10 pfu per cell of either Ad-GFP plus Ad-Trans or Ad-IE86 plus Ad-Trans and incubated for 48 h. **(A)** Cells were treated with DMSO (vehicle for MG132 and epoxomicin, lanes 1 and 4), MG132 (lanes 2 and 5), epoxomicin (lanes 3 and 6), ddH_2_O (vehicle for chloroquine, lanes 7 and 9) or chloroquine (lanes 8 and 10). At 12 h after treatment, cells were harvested, and equal amounts of cell extracts were subjected to western blot analysis with antibodies to STING, HCMV IE86, LC3B and tubulin. **(B)** Cells were pre-treated with MG132 for 12 h and followed by a cycloheximide chase for the indicated time points. Equal amounts of cell extracts were subjected to western blot analysis with antibodies to STING, TBK1, HCMV IE86 and tubulin. The signal intensity of protein bands was analyzed using Image Lab^TM^ software for determining relative protein levels of STING at the indicated chase time points. Epox, epoxomicin; CQ, chloroquine; DW, ddH_2_O; CHX, cycloheximide.

To determine whether IE86 protein induces STING degradation via a proteasome- or lysosome-dependent pathway, cells were treated with either proteasome inhibitors, MG132 and epoxomicin, or a lysosome inhibitor, choloroquine (**Figure 6A**). Both MG132 or epoxomicin treatment restored the level of STING protein in Ad-IE86-transduced cells similar to that in Ad-GFP-transduced cells, indicating that IE86 induces proteasome-dependent degradation of STING protein (**Figure 6A**, compare lanes 5 and 6 with 4). Although microtubule-associated protein light chain 3 (LC3) isoform B (LC3B) was significantly accumulated in cells treated with chloroquine, the level of STING protein was restored to a lesser extent than in cells treated with proteasome inhibitors (**Figure 6A**, compare lane 10 with 9). Thus, IE86 protein induces STING degradation mainly via a proteasome-dependent pathway.

Since IE86 protein reduced the steady-state protein level of STING, the effect of IE86 protein on the half-life of STING protein was determined using a pulse-chase experiment (**Figure 6B**). Cells transduced with either Ad-GFP or Ad-IE86 were pretreated with MG132 for 12 h and followed by a cycloheximide chase for the indicated time points. In Ad-GFP-transduced cells, the endogenous STING protein half-life was approximately 50 min. On the other hand, the half-life of STING protein was substantially reduced (approximately < 25 min) in cells transduced with Ad-IE86 (**Figure 6B**). The half-life of TBK1 protein was reported to be approximately 2.5 h in NIH3T3 cells (Sun et al., 2014). In HFF cells, the approximate half-life of TBK1 protein was 4 h, and IE86 protein had no additive effect on the stability of TBK1 protein (**Figure 6B**). These data further support the hypothesis that IE86 protein promotes the proteasome-dependent degradation of STING protein.

### IE86 Protein Interferes with 2′3′-cGAMP-Induced IFN-β Promoter Activation and ISG Expression

**FIGURE 7:**
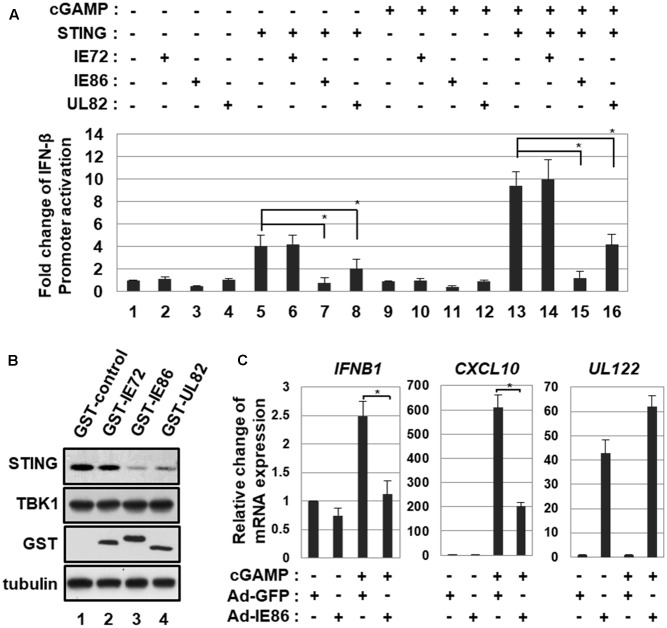
HCMV IE86 protein interferes with 2′3′-cGAMP-induced signaling pathway. **(A)** HEK293T cells were co-transfected with small amounts of vectors expressing STING and control *renilla* luciferase reporter and IFN-β promoter-driven firefly luciferase reporter plasmids plus the expression vector for IE72, IE86 or UL82. At 18 h after transfection, cells were treated with a STING agonist, 2′3′-cGAMP, and IFN-β promoter-driven luciferase activities were determined at 12 h after treatment. **(B)** HEK293T cells were transfected with the vector expressing STING plus the expression vector for GST, GST-IE72, GST-IE86 or GST-UL82. At 24 h after transfection, cells were harvested, and equal amounts of cell extracts were subjected to western blot analysis with antibodies to STING, TBK1, GST and tubulin. **(C)** HFF cells were transduced with 10 pfu per cell of either Ad-GFP plus Ad-Trans or Ad-IE86 plus Ad-Trans. At 48 h after transduction, cells were treated with 2′3′-cGAMP, and relative amounts of *IFNB1*, *CXCL10* and *UL122* transcripts were measured using qRT-PCR at 12 h after treatment. The data shown here represent three independent experiments ±*SD*. The asterisk (^∗^) denotes a significant difference between samples, which was determined by the *P*-value of a two-sample *t*-test (*P* < 0.05).

To determine whether HCMV IE86 protein interferes with STING-induced type I IFN pathway, HEK293T cells were co-transfected with small amounts of vectors expressing STING and control *renilla* luciferase reporter and IFN-β promoter-driven firefly luciferase reporter plasmids plus the expression vector for IE72, IE86 or UL82 and then treated with a STING agonist, 2′3′-cGAMP. In human cells, 2′3′-cGAMP is produced by cGAS and binds to STING to induce type I IFN pathway (Ablasser et al., 2013; Civril et al., 2013). At 12 h after treatment, IFN-β promoter-driven luciferase activities were measured (**Figure 7A**). Since HEK293T cells do not express detectable STING (Diner et al., 2013), 2′3′-cGAMP treatment had no effect on IFN-β promoter activation (**Figure 7A**, compare lane 9 with 1). Ectopic expression of STING in these transfection settings enhanced IFN-β promoter activity 4-fold, and 2′3′-cGAMP treatment further activated to 9.4-fold (**Figure 7A**, compare lanes 5 and 13 with 1). In HEK293T cells expressing ectopic STING protein, both IE86 and UL82 proteins suppressed 2′3′-cGAMP-induced IFN-β promoter activity by 87 and 56%, respectively (**Figure 7A**, compare lanes 15 and 16 with 13). The IE72 protein had no effect on 2′3′-cGAMP-induced IFN-β promoter activation (**Figure 7A**, compare lane 14 with 13). Both IE86 and UL82, but not IE72, proteins significantly reduced the level of STING protein (**Figure 7B**, compare lanes 3 and 4 with 1).

To further determine the effect of IE86 protein on 2′3′-cGAMP-induced ISG expression, HFF cells transduced with Ad-GFP or Ad-IE86 were treated with 2′3′-cGAMP, and levels of ISGs such as *IFNB1* and *CXCL10* were determined using qRT-PCR at 12 h after treatment. In cells transduced with Ad-GFP, 2′3′-cGAMP treatment induced levels of *IFNB1* and *CXCL10* 2.5- and 610-folds, respectively (**Figure 7C**). The expression of IE86 protein significantly reduced 2′3′-cGAMP-induced levels of *IFNB1* and *CXCL10* by 56 and 67%, respectively (**Figure 7C**). Thus, these results suggest that HCMV IE86 protein blocks STING-induced signaling pathway.

### IE86 Protein Inhibits STING-Induced TBK1 Activation

**FIGURE 8:**
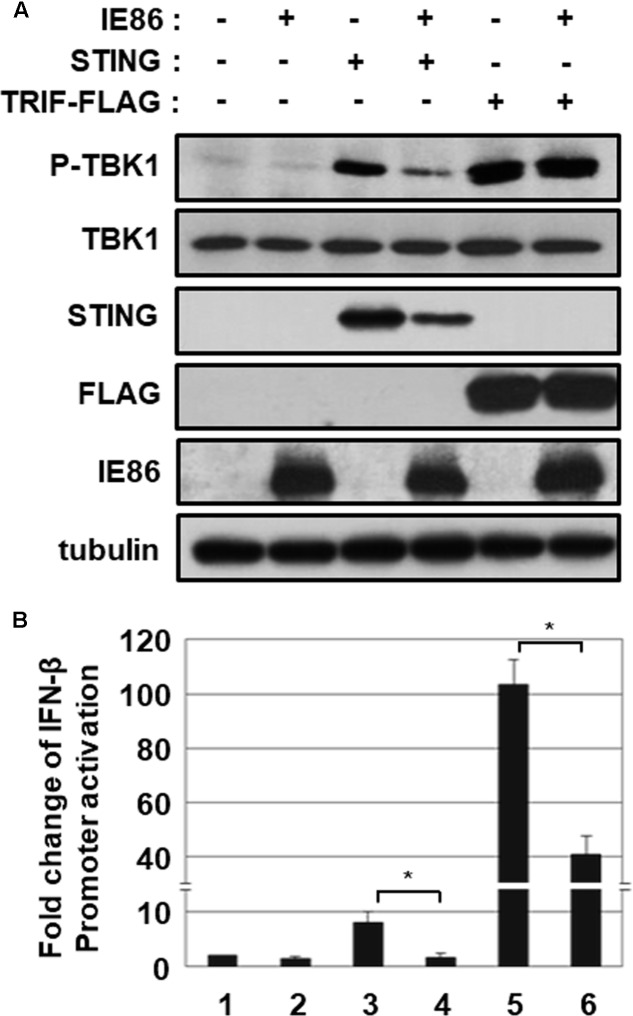
IE86 protein inhibits STING-induced TBK1 activation. **(A)** HEK293T cells were transfected with the vector expressing STING or TRIF with control or IE86 expression vector. At 24 h after transfection, equal amounts of cell extracts were subjected to western blot analysis with antibodies to phospho-TBK1, TBK1, STING, FLAG, HCMV IE86 and tubulin. **(B)** HEK293T cells were co-transfected with the vector expressing STING or TRIF, control *renilla* luciferase reporter and IFN-β promoter-driven firefly luciferase reporter plasmids plus control or IE86 expression vector. At 48 h, IFN-β promoter-driven luciferase activity was measured using a dual luciferase assay system. IFN-β promoter-driven luciferase activity was expressed in RLU by normalizing firefly luciferase activity with constitutive *renilla* luciferase activity. Luciferase data shown here represent three independent experiments ±*SD*. The asterisk (^∗^) denotes a significant difference between samples, which was determined by the *P*-value of a two-sample *t*-test (*P* < 0.05).

To ascertain the effect of IE86 protein-mediated degradation of STING protein on STING-induced signaling pathway, HEK293T cells were transfected with the vector expressing STING or Toll/IL-1 receptor (TIR)-domain-containing adaptor-inducing IFN-β (TRIF), and TBK1 phosphorylation at serine 172, indicative of TBK1 activation, was determined (**Figure 8A**). In HEK293T cells, overexpression of STING or TRIF protein induced phosphorylation of TBK1 at serine 172 (**Figure 8A**, compare lanes 3 and 5 with 1). IE86 protein had no effect on both the level of TRIF protein and TRIF-induced TBK1 activation (**Figure 8A**, compare lanes 6 with 5). However, the level of STING protein and STING-induced TBK1 activation were significantly reduced in cells expressing IE86 protein (**Figure 8A**, compare lane 4 with 3).

To further determine whether IE86 protein affects STING- or TRIF-induced IFN-β promoter activation, the IFN-β promoter-driven luciferase reporter assay was performed (**Figure 8B**). Ectopic expression of STING and TRIF strongly activated IFN-β promoter-driven luciferase activity 7.9- and 103-folds, respectively (**Figure 8B**, lanes 3 and 5). IE86 protein significantly reduced both STING- and TRIF-induced IFN-β promoter activation, possibly by inhibiting NF-κB activity as previously reported (**Figure 8B**, compare lanes 4 and 6 with 3 and 5) (Taylor and Bresnahan, 2005, 2006). Taken together, these data support the hypothesis that IE86 protein interferes with STING-induced signaling pathway by down-regulating the level of STING protein and inhibiting transcription factors for IFN-β promoter activation.

### Determination of the IE86 Protein Residues Responsible for STING Degradation

**FIGURE 9:**
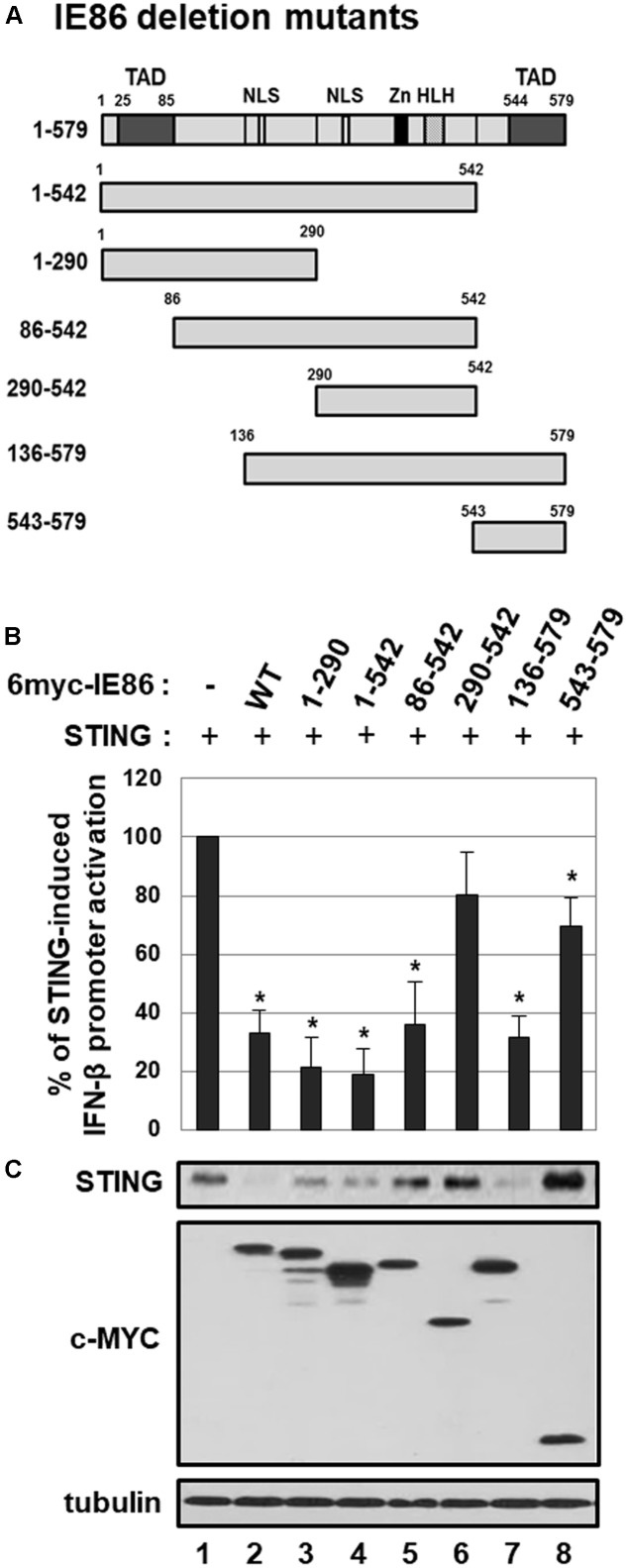
The effect of IE86 mutants on STING. **(A)** A schematic diagram of IE86 deletion mutants. TAD, transactivation domain; Zn, zinc finger domain; HLH, helix-loop-helix motif; NLS, nuclear localization sequence **(B)** HEK293T cells were co-transfected with the vector expressing STING, control *renilla* luciferase reporter and IFN-β promoter-driven firefly luciferase reporter plasmids plus pCS3-MT vector expressing 6X-myc-tagged IE86 WT or deletion mutants. At 48 h, IFN-β promoter-driven luciferase activity was measured using a dual luciferase assay system. IFN-β promoter-driven luciferase activity was expressed in RLU by normalizing firefly luciferase activity with constitutive *renilla* luciferase activity. To determine the effect of IE86 WT or deletion mutants on STING-induced IFN-β promoter activation, RLU in cells co-transfected with vectors expressing STING and IE86 WT or deletion mutants was divided by that in cells co-transfected with control vector and the vector expressing IE86 WT or deletion mutants. To analyze the relative luciferase activity, STING-induced luciferase activities without the vector expressing IE86 WT or deletion mutants were set to 100%. Luciferase data shown here represent three independent experiments ±*SD*. The asterisk (^∗^) denotes a significant difference between control and IE86 (WT or deletion mutants) expressing samples, which was determined by the *P*-value of a two-sample *t*-test (*P* < 0.05). **(C)** HEK293T cells were co-transfected with vectors expressing STING and 6X-myc-tagged IE2 WT or deletion mutants. At 48 h after transfection, equal amounts of cell extracts were subjected to western blot analysis with antibodies to STING, c-Myc and tubulin.

To further elucidate the IE86 protein residues that are required for inhibition of STING-induced IFN-β promoter activation and down-regulation of STING protein levels, HEK293T cells were co-transfected with expression vectors for STING and myc-tagged IE86 WT or deletion mutants (**Figure 9A**). At 48 h after transfection, IFN-β promoter activities and the level of STING protein were assessed by luciferase reporter assay and western blot analysis, respectively (**Figures 9B,C**). Expression of full-length or truncated IE86 protein containing amino acids (aa) 1-290, 1-542, 86-542 or 136-579 significantly reduced STING-induced IFN-β promoter activation (**Figure 9B**). On the other hand, expression of IE86 mutant protein containing aa 290–542 or 543–579 failed to exhibit significant inhibitory effect on STING-induced IFN-β promoter activation (**Figure 9B**). Expression of full-length or truncated IE86 protein containing aa 1–290, 1–542, or 136–579 significantly reduced the level of STING protein in HEK293T cells (**Figure 9C**, compare lanes 2, 3, 4 and 7 with 1). However, expression of IE86 mutant protein containing aa 86–542, 290–542, or 543–579 failed to reduce the level of STING protein in HEK293T cells (**Figure 9C**, compare lanes 5, 6 and 8 with 1). Taken together, aa 1-289 of the IE86 protein may be critical for down-regulating both STING-induced IFN-β promoter activation and the level of STING protein.

## Discussion

The type I IFN pathway plays an important role in limiting HCMV replication. Pre-infection or post-infection treatment of type I IFN significantly inhibits virus production (Delannoy et al., 1999; Sainz et al., 2005; Taylor and Bresnahan, 2005). Furthermore, type I IFN deficiency enhances the rate of HCMV replication and spread in human fibroblasts (McSharry et al., 2015).

DNA sensors such as DNA-dependent activator of IFN-regulatory factors (DAI), IFI16 or cGAS were reported to detect HCMV infection to induce type I IFN activation (DeFilippis et al., 2010; Gariano et al., 2012; Li et al., 2013; Lio et al., 2016; Paijo et al., 2016). Since CRISPR/Cas9 KD of IFI16 has no effect on HCMV-induced TBK1 activation, IFI16 is not required for STING-TBK1-IRF3 activation in human fibroblasts upon HCMV infection (Diner et al., 2016). Nonetheless, IFI16 still limits HCMV replication possibly by inducing antiviral cytokines or utilizing an unknown mechanism(s). The cGAS-STING pathway may play an important role in the type I IFN pathway upon HCMV infection because KD of either cGAS or STING strongly abolishes activation of TBK1 and IRF3 (Diner et al., 2016; Paijo et al., 2016).

In HFF cells stably transduced with pLKO.1-STING shRNA, the HCMV titer was increased 4.3-fold (**Figure 1A**), whereas STING KD exhibited more profound effect on HCMV infection in HUVEC cells (9.9-fold increase in viral titer at 7 days after infection) (Lio et al., 2016). These discrepancies may be explained by the different infection efficiency and/or methods used to KD STING (CRISPR/Cas9 versus shRNA). In addition, other antiviral signaling pathways and/or restriction factors including IFI16 may also contribute to the inhibition of HCMV replication in HFF cells.

Previous reports indicate that HCMV infection down-regulates protein levels of JAK1, STAT2 and HLA-DR (Miller et al., 1998; Le Roy et al., 1999; Le et al., 2008), and a quantitative proteomic analysis in HFF cells indicates that HCMV infection initially induces the expression of ISGs but progressively reduces levels of proteins involved in the type I IFN pathway including RIG-I, STING, IRF3, Jak1, STAT2 and IRF9 (Weekes et al., 2014). Down-regulation of STING protein level in HCMV-infected HFF cells was consistent with the proteomics data (**Figure 4A**) (Fu et al., 2017). However, HCMV infection has no effect on the protein level of STING in plasmacytoid dendritic cells (pDC), monocyte-derived dendritic cells (moDC) or granulocyte-macrophage colony-stimulating factor (GM-CSF) cultured bone marrow-derived macrophages (GM-CSF-MΦ), whereas it slightly reduces the protein level of STING in monocyte-derived macrophages (moMΦ) (Paijo et al., 2016). The differences in the efficiency of infection or viral gene expression may contribute to the discrepancy in STING protein levels between cell types upon HCMV infection.

Given the importance of STING in the innate immune response against DNA viruses, it is not surprising that 7 viral genes (UL25, UL36, UL82, UL89B, UL94, UL122 and US23) have been identified in HCMV cDNA library screening to significantly down-regulate the STING-induced IFN-β promoter activation. Although additional enhancement of STING-induced IFN-β promoter by UL76 and UL50 is noteworthy, the importance of IFN-β promoter enhancement by these viral proteins in the context of HCMV life cycle is unclear. HCMV UL76 protein was reported to induce IL-8 expression via NF-κB activation (Costa et al., 2013). Thus, STING-induced IFN-β promoter activation was further enhanced by UL76 possibly through NF-κB activation. For the screening, we used HEK293T cells which do not express detectable STING protein to avoid the effect of endogenous STING protein. Although SV40 large T antigen was reported to antagonize the cGAS-STING pathway (Lau et al., 2015), ectopic expression of STING overrode it and restored IFN-β promoter activation in HEK293T cells (**Figures 3, 7A**).

Initially, we decided to study HCMV UL122 encoding IE86 protein because it (i) reduced STING-induced IFN-β promoter activation most effectively among the screened HCMV ORF (**Figure 3**) and (ii) strongly interacted with STING in the yeast two hybrid screening to identify STING-interacting HCMV ORFs (**Figure 4**). Given the fact that IE86 protein is predominantly localized in the nucleus, it was unexpected that IE86 protein interacts with STING in the yeast two-hybrid assay. In addition, we were unable to determine the interaction between IE86 and STING in HCMV-infected HFF cells with or without MG132 treatment (data not shown). Since MG132 affects the MIE promoter and/or may induce adverse effects on cells (DeMeritt et al., 2004), it is still unclear whether STING directly binds to IE86 during HCMV infection.

In accordance with the previous report (Fu et al., 2017), UL82 protein was also identified to inhibit STING-induced IFN-β promoter activation in our screening (**Figure 3**). Since UL82 is critical for efficient expression of IE genes (Bresnahan and Shenk, 2000), inefficient expression of IE86 protein may affect enhanced expression of *IFNB1*, *ISG56* and *TNF* transcripts by HCMV in HFF cells with stable KD of UL82 (Fu et al., 2017). Although UL82 protein was reported to bind STING (Fu et al., 2017), it failed to interact with STING in our yeast two-hybrid screening (**Figure 4**). Therefore, interaction between UL82 protein and STING may be indirect and involve other cellular proteins such as iRhom2 (Fu et al., 2017).

The data in **Figure 6** indicate that IE86 protein induces the proteasome-dependent degradation of STING. Since IE86 protein is an essential transactivator for both viral and cellular genes, it is possible that cellular machineries activated by IE86 protein facilitate degradation of STING. E3 ubiquitin ligases such as RNF5 (RMA1) and tripartite motif protein 30α (TRIM30α) catalyze lysine 48-linked polyubiquitination and proteasome-dependent degradation of STING (Zhong et al., 2009; Wang et al., 2015). However, IE86 protein had no effect on the expression of RNF5 and TRIM30α (data not shown). Whether IE86 protein directly facilitates degradation of STING or indirectly activates an E3 ubiquitin ligase(s) for STING is unclear and is a subject of future investigation.

Our data suggest that IE86 protein can interfere with STING-induced signaling pathway by down-regulating the level of STING protein as well as inhibiting transcription factors for IFN-β promoter activation. Domain mapping using deletion mutants of the IE86 protein reveals that aa 1–289 are required for inhibiting STING-induced IFN-β promoter activation, and both aa 1–85 and 136–289 are critical for promoting STING degradation (**Figure 9**). The IE86 aa 136 to 290 region is rich in serine and threonine residues which is highly phosphorylated and is critical for interaction with various host factors such as Retinoblastoma Protein (Rb) or TATA-binding protein (TBP) (Sommer et al., 1994). HPV E7 and adenovirus E1A proteins inhibit the cGAS-STING DNA sensing pathway by using the LXCXE motif which is essential for Rb binding (Lau et al., 2015). Although the Rb family of proteins is dispensable for antagonizing the cGAS-STING pathway by the oncogenes of the DNA tumor viruses (Lau et al., 2015), the functional connection between the cell cycle and STING regulation still needs to be addressed. Interestingly, expression of IE86 mutant protein containing aa 86–542 reduced STING-induced IFN-β promoter activation but failed to down-regulate the level of STING protein (**Figure 9**). Since IE86 protein regulates gene expression by interacting with numerous viral and cellular proteins (reviewed in Stinski and Petrik, 2008), it is possible that the IE86 mutant protein containing aa 86–542 attenuates transcription factors for IFN-β promoter activation and inhibits STING-induced IFN-β promoter activation without promoting degradation of STING protein. Indeed, the IE86 mutant protein containing aa 86–542 interfered with TRIF-induced IFN-β promoter activation (data not shown). Therefore, IE86 protein may possess two independent functions in facilitating degradation of STING protein and inhibiting cellular transcription factors for IFN-β promoter activation to interfere with the STING signaling pathway.

## Author Contributions

J-EK and Y-JS designed experiments, analyzed data, and wrote the manuscript. J-EK and Y-EK performed experiments. MS and J-HA provided materials. Y-JS, MS, and J-HA revised the manuscript.

## Conflict of Interest Statement

The authors declare that the research was conducted in the absence of any commercial or financial relationships that could be construed as a potential conflict of interest.

## Abbreviations

cGAMP: cyclic GMP-AMP
HCMV: human cytomegalovirus
IE: immediate-early
IFN: interferon
STING: stimulator of interferon genes
ORF: open reading frame
