# Corrigendum: Human Cytomegalovirus IE2 86 kDa Protein Induces STING Degradation and Inhibits cGAMP-Mediated IFN-β Induction

**DOI:** 10.3389/fmicb.2018.00350

**Published:** 2018-02-26

**Authors:** Jung-Eun Kim, Young-Eui Kim, Mark F. Stinski, Jin-Hyun Ahn, Yoon-Jae Song

**Affiliations:** ^1^Department of Life Science, Gachon University, Seongnam, South Korea; ^2^Department of Molecular Cell Biology, Samsung Biomedical Research Institute, Sungkyunkwan University School of Medicine, Suwon, South Korea; ^3^Department of Microbiology, Carver College of Medicine, University of Iowa, Iowa City, IA, United States

**Keywords:** HCMV, IE86, STING, IFN, cGAMP

In the published article, there was an error in affiliations 2 and 3. Instead of “^2^Department of Microbiology, Carver College of Medicine, University of Iowa, Iowa City, IA, United States” and ^“3^Department of Molecular Cell Biology, Samsung Biomedical Research Institute, Sungkyunkwan University School of Medicine, Suwon, South Korea”, it should be “^2^Department of Molecular Cell Biology, Samsung Biomedical Research Institute, Sungkyunkwan University School of Medicine, Suwon, South Korea” and “^3^Department of Microbiology, Carver College of Medicine, University of Iowa, Iowa City, IA, United States”. The authors apologize for this error and state that this does not change the scientific conclusions of the article in any way.

The original article has been updated.

## Conflict of interest statement

The authors declare that the research was conducted in the absence of any commercial or financial relationships that could be construed as a potential conflict of interest.

